# Acute Myeloid Leukemia with Myelodysplasia-Related Gene Mutations

**DOI:** 10.3390/jcm15155958

**Published:** 2026-07-30

**Authors:** Ugo Testa

**Affiliations:** Department of Oncology, Istituto Superiore di Sanità, Viale Regina Elena 299, 00161 Rome, Italy; ugotesta@gmail.com

**Keywords:** acute myeloid leukemia, myelodysplasia, leukemia classification, gene sequencing, prognostic stratification, cytogenetics

## Abstract

**Background/Objectives**: Acute myeloid leukemia (AML) with myelodysplasia-related gene mutations (AML-MR), often referred to as secondary AML (s-AML) or AML-MRC, is an aggressive form of leukemia that typically arises from an antecedent myelodysplastic syndrome (MDS) but may also originate de novo. It is characterized by mutations in key genes associated with MDS that include some epigenetic regulators (*ASXL1* and *EZH2*), splicing factors (*SF3B1*, *SRSF2*, *U2AF1*, and *ZRSR2*), and transcription factors (*BCOR*, *RUNX1*, and *STAG2*). The main objective of this review paper consists of analyzing recent studies that have improved the criteria for the characterization, definition, and classification of AML-MR. **Methods**: An extensive search of the most recent literature on the topic was performed, selecting and critically analyzing the most relevant studies. **Results**. The studies carried out in the last few years have provided an extensive molecular characterization of AML-MR, supporting sounder criteria for identification and a better definition with respect to other AML subtypes, particularly with respect to *TP53*-mutant AML. **Conclusions**: A unifying classification of AML-MR is now possible, allowing its identification as a unique, well-defined, and separate entity.

## 1. Introduction

The World Health Organization (WHO) classification of myeloid neoplasms was revised several times to provide a progressively more accurate understanding of the cellular and molecular features underlying these tumors. The development of techniques allowing the exploration of the human genome has permitted a more accurate definition of the molecular abnormalities occurring in myeloid neoplasms. In particular, these studies have shown the extensive molecular heterogeneity of acute myeloid leukemias (AMLs), allowing their classification into molecular subtypes that are distinguished from their genetic alterations, prognosis, and response to therapy. The wide diffusion of techniques of gene analysis, such as next-generation sequencing, has permitted the introduction of gene information in the diagnostic criteria for AML. Several recent studies have provided a detailed analysis of the genetic abnormalities occurring in AML and a detailed census of the genes altered by structural changes or mutational events in AML [1,2,3].

The World Health Organization (WHO) classification of AML proposed in 2016 classifies AML into four main subtypes: AML with recurrent genetic abnormalities, AML with myelodysplasia-related changes (AML-MRC), therapy-related AML (t-AML), and AML not otherwise specified [4]. The diagnostic criteria for AML-MRC (WHO 2016) are the following: history of myelodysplastic syndrome or MDS/myeloproliferative neoplasms, morphologic dysplasia in >50% of cells of at least two lineages, and the presence of MDS-defining cytogenetic abnormalities [4]. Since the evaluation of dysplasia appeared subjective, with limited interobserver agreement, its value was questioned, and the ontogeny of AML outweighed genomics; new classifications have introduced new criteria for the diagnosis of AML-MRC.

Thus, data on gene abnormalities have been introduced into AML diagnosis and have contributed to a more accurate risk stratification. This molecular information has been incorporated into the recent molecular and prognostic classifications of AMLs. In particular, the fifth edition of the WHO classification (2022 WHO) and the International Consensus Classification (ICC) of myeloid neoplasms were published in 2022 [5,6]. Notable changes introduced into these classifications include the lowering of the blast threshold that defines AML and the renaming of myelodysplastic syndrome and myelodysplastic neoplasm. An additional and very important change introduced into the WHO/ICC 2022 classifications with respect to the fourth WHO classification (2016 WHO) concerned the criteria for the definition and classification of the AML subset associated with myelodysplasia. As discussed above, in the 2016 WHO guidelines, the main criteria for the diagnosis of AML with myelodysplasia-related changes (AML-MRC) are represented by morphological features in the bone marrow, a history of MDS, and chromosomal abnormalities typical of MDS [4]. In the 2022 WHO publication, the criteria for the diagnosis of AML-MRC changed, and morphological dysplasia alone was excluded from the diagnostic criteria, while mutations in at least one of these myelodysplasia-related genes (*ASXL1*, *BCOR*, *EZH2*, *SF3B1*, *SRSF2*, *STAG2*, *U2AF1*, and *ZRSR2*) were included [5]. These genes include three splicing modulators (*SF3B1*, *SRSF2*, and *U2AF1*), three epigenetic regulators (*ASXL1*, *EZH2*, and *BCOR*) and two transcription factors (*RUNX1* and *STAG2*). In the 2022 ICC guidelines, the criteria for AML-MRC also included additional *RUNX1* mutations to the myelodysplasia-related genes (MRG) [6] (Table 1).

An additional element of discrepancy between the 2022 classifications pertains to the biological boundaries between MDS and AML, with the new MDS-AML category introduced by ICC defined by 10–19% blasts; this group largely overlaps with MDS in terms of excess blasts 2 [6]. Furthermore, a previous history of MDS was considered among the diagnostic criteria for WHO 2022 and a qualifier for ICC 2022 (Table 1).

In the studies on AML-MR, two groups of patients can be distinguished: AML with MRG mutations and without MR-chromosome abnormalities, and AML with MR chromosome abnormalities, such as chromosome 5 and 7 abnormalities; each of the groups can be subdivided according to clinical history, in that some patients have developed AML post-MDS or -MDS/MPN. The AML category, with MDR cytogenetic abnormalities, incorporates cases with a complex karyotype (defined as ≥3 unrelated chromosomal abnormalities) and/or other unbalanced chromosomal changes that previously fell into the prior AML-MRC category but lack a *TP53* mutation, MDR gene mutations, or chromosome 5, 7, 17, or 20 abnormalities.

In many of the current studies, five groups of AML patients are defined:

- De novo AML: newly diagnosed AML without a clinical history of an antecedent hematological disorder; after the establishment of the genome signature, AML patients with class-defining mutations, such as patients with *NPM1* mutations, are assigned to the de novo AML category irrespective of the presence of MRG mutations;
- S-AML: clinically defined AML preceded by clinically documented MDS;
- MDS/AML-MRG: molecularly defined secondary-type MDS/AML with at least one mutation of *ASXL1*, *BCOR*, *EZH2*, *RUNX1*, *SF3B1*, *SRSF2*, *STAG2*, *U2AF1*, and *ZRSR2* (10–19% of blasts);
- AML-MRG: molecularly defined secondary-type AML with at least one mutation of *ASXL1*, *BCOR*, *EZH2*, *RUNX1*, *SF3B1*, *SRSF2*, *STAG2*, *U2AF1*, and *ZRSR2* (>20% of blasts);
- *TP53*-mutant AML with mutations in *TP53* and/or chromosome abnormalities involving chromosome 17p.

## 2. Aim and Methodology

The present study is based on a systematic review of the current literature on AML-MR. The aim of the present review paper was to critically analyze and discuss recent studies involved in the molecular characterization of AML-MR.

This study was based on a literature search of the PubMed database, using the terms acute myeloid leukemia, acute myeloid leukemia with myelodysplasia changes, acute myeloid leukemia classification, myelodysplasia, NPM1-mutant AML, FLT3-mutant AML, and TP53-mutant AML. The search period was expanded from 1990 to 2026. The inclusion criteria for the screened literature were limited to studies published in peer-reviewed publications in high-impact journals in hematology. Only experimental or clinical studies based on large cohorts of patients were included.

Furthermore, the Google search engine was used to find the link to major recent international hematology and oncology meetings, which were screened for congress abstracts related to AML-MR.

## 3. Molecular and Clinical Features of AML-MR

Several studies have shown that the frequency of MRG mutations greatly increases with age. Hoff and coworkers reported the molecular characterization of 1023 AML patients aged >60 years (median age of 72 years) enrolled in the context of the BEAT study. In this group of patients, three MR genes were among the most mutated: *RUNX1* (22%), *SRSF2* (22%), and *ASXL1* (21%) [7]. Globally, MRGs were mutated in 57% of these patients, which is more than twice the frequency in younger patients [8]. Cusan et al. have analyzed the mutational and cytogenetic profiles in AML patients of different ages, from 18–10 years up to >75 years; some gene mutations, such as those involving *RUNX1*, *SRSF2*, *ASXL1*, and *BCOR*, display a strong age-dependent pattern, thus explaining the marked rise of the frequency of MRG mutations observed in older AML patients [9]. Jahn et al. have explored the genomic landscape and leukemogenic pathways in a cohort of 815 older AML patients treated in the context of the ASTRAL-1 trial [10]. NGS studies showed frequent mutations in many MR genes, such as *ASXL1*, *SRSF2*, and *RUNX1* [10]. To delineate leukemia-initiating trajectories, an oncogenetic tree was constructed by inferring the sequence of mutation acquisition and exploring the existing relationship between genetic alterations; the elaborated algorithm defined an oncogenetic tree with five branches, with *ASXL1*, *DDX41*, *DNMT3A*, *SRSF2*, and *TET2* emanating from the root. Importantly, the tree originating from the *ASXL1* node gives rise to several individual clones, with *EZH2*, *NRAS*, *RUNX1*, *SRSF2*, and *U2AF1*; *BCOR*, *JAK2*, *KRAS*, *PHF6*, and *SF3B1* originating from *RUNX1*; and *STAG2* from the *SRSF2* node. Importantly, the branches originating from the *ASXL1* node include eight of the nine genes defining the AML-MR category [10]. Clustering by hierarchical Dirichlet processes identified five distinct groups of AML; importantly, the largest group, class 1 (49%), was characterized by the presence of the nine genes defining AML, with MRG mutations and MR cytogenetic abnormalities [10].

A key study by Lindsley and coworkers described the basic features of AML-MRC [11]. These authors showed that the presence of *SRSF2*, *SF3B1*, *U2AF1*, *ZRSR2*, *ASXL1*, *EZH2*, *BCOR*, or *STAG2* identified a subgroup of AMLs, defined as secondary AML (s-AML); analysis of serial samples from individual patients showed that these mutations occur early in leukemogenesis and persist in clonal remissions [11]. Patients with MRG mutations were older, leukopenic at diagnosis, and had a worse outcome overall [11].

Several studies have explored the prognostic impact of MRG mutations and have contributed to understanding their prognostic heterogeneity, partly related to their association or lack thereof with favorable co-mutations.

Several retrospective studies involving large cohorts of intensively treated patients have confirmed the unfavorable prognostic impact of MRG mutations [12,13].

Zhou and coworkers reported a real-world retrospective analysis of clinical outcomes in AML with MR; this analysis compared the WHO-2022 criteria for AML-MR with the ICC-2022 criteria for AML-MR [14]. The clinicopathological features were similar, except for higher rates of complex karyotypes, monosomy 17, *TP53* mutations, and fewer *RUNX1* mutations in the WHO-AML-MR group [14]. AML-MRs classified in both classification systems had inferior outcomes compared to AML without MR, and better outcomes than *TP53*-mutated AMLs [10]. ICC-AML-MRC had better outcomes than WHO-AML-MRC, but this is due to the classification of *TP53*-mutant AML as a separate group in the ICC-AML classification.

As mentioned above, the ICC 2022 classification excludes *TP53*-mutant AML from the AML-MR category. The rationale is that *TP53* mutations drive a uniquely aggressive, distinct disease biology and marked chemoresistance that supersede typical myelodysplasia features. *TP53*-mutant AML presents a distinct genomic profile compared to AML-MR, a survival profile that is poorer than AML-MR, and a different profile of response to treatments, with marked resistance to standard induction chemotherapy and to hypomethylating agents plus venetoclax.

Recent studies have explored the mechanisms responsible for the variable prognostic impact of MRG mutations.

As MRG mutations are acquired at an early stage of disease evolution and further mutations are acquired over the course of disease, the clonality of mutations could provide a better understanding of the prognostic impact of MRG mutations. Thus, Mecklenbrauck and coworkers have explored the prognostic impact of the clonal representation of MRG mutations in a group of 550 AML-MRC patients [15]. These patients were stratified into three risk groups according to 2022 ELN criteria: favorable risk (20%), intermediate risk (5%), and adverse risk (75%). Patients with a favorable-risk profile had similar EFS and OS with or without MRG mutations [15]. Patients with MRG mutations with an adverse-risk profile had similar EFS but longer OS compared to the rest of the adverse-risk AML patients without MRG mutations [15]. Patients with MRG mutations were subdivided into two groups according to the variant allele frequency (VAF) of MRG mutant genes: low VAF (<44.5%) and high VAF (>44.5%). High-VAF patients were significantly older and had a high WBC count and lower platelet count at diagnosis. *ASXL1* mutations were more likely to be mutated in the high-VAF group, while *SF3B1* and *STAG2* mutations were predominantly observed in the low-VAF group. High-VAF patients showed a significantly shorter EFS and OS compared to low-VAF patients, and patients with a high VAF had a higher cumulative incidence of relapse compared to the low-VAF group [15]. Among patients with a favorable-risk profile, MRG VAF did not affect prognosis; in contrast, in the adverse-risk group, patients with a high MRG mutation VAF had significantly shorter EFS and OS compared to those with a low VAF [15]. These observations support the hypothesis that MRG mutations drive the more aggressive phenotype of leukemic cells when they are part of the dominant clone [15].

A recent study explored the differential prognostic impact of MRG mutations in a large cohort of 4978 AML patients who were molecularly characterized and intensively treated [16]. A total of 1698 of these patients (34.1%) harbor MRG mutations at the level of at least one gene. The MR genes most frequently mutated were *RUNX1* (12.8%), *ASXL1* (9.9%), *SRSF2* (9.2%), *STAG2* (5.9%), *BCOR* (5.4%), and *EZH2* (4.1%) [16]. AML-MR cases displayed a lower CR rate (65.7% vs. 77%), shorter EFS (6.3 months vs. 10.5 months), RFS (14.3 months vs. 20.5 months), and OS (16.6 months vs. 26.5 months) compared to AMLs without MRG mutations [16]. Outcomes were also explored in a subgroup of patients who underwent allo-HSCT in CR1: compared to patients without MRG mutations, patients with MRG mutations showed a shortened EFS (26.5 months vs. 50.5 months), RFS (41.1 months vs. 101.9 months), and OS (63.4 months vs. not reached) [16]. Analysis of patient outcomes with respect to individual MRG mutations showed three groups: patients with *ASXL1*, *RUNX1*, *SF3B1*, or *U2AF1* mutations had significantly shorter median EFS (event-free survival) than patients in the ELN intermediate risk group, with no significant differences compared to the adverse risk group; patients with alterations in *SRFSF2* or *STAG2* mutations showed no survival difference compared to the ELN 2022 intermediate risk patients, but a significantly longer EFS than adverse risk patients; patients with *BCOR*, *EZH2*, and *ZRSR2* mutations had an EFS intermediate between the intermediate or adverse risk groups, with no statistically significant differences compared to either risk group [16]. Regarding OS, patients with *ASXL1*, *RUNX1*, *SF3B1*, and *U2AF1* mutations were associated with adverse risk; *BCOR*, *SRSF2*, and *STAG2* were aligned with intermediate risk; and *ASXL1*, *EZH2*, and *ZRSR2* did not significantly differ from intermediate or adverse risk groups [16].

It is important to note that the prognostication of various MRG mutations was established based on the response to intensive chemotherapy treatment. The prognostic impact of SF mutations has been evaluated in a group of 994 patients with newly diagnosed (ND) AML, including 266 patients with a SF mutation (*SRSF2*, *U2AF1*, *SF3B1*, and *ZRSF2*), with a median age of 67 years; in patients treated with IC, the median OS was significantly shorter for SF-mutant patients than for SF-WT patients (15.9 vs. 26.7 months); this significance was abrogated when evaluating patients who received Ventoclax (VEN) with intensive chemotherapy (IC, mOS of 19.6 vs. 30.7 months) or VEN with low-intensity therapy (mOS of 12.3 vs. 8.5 months) [17]. These observations were supported by experimental studies showing a unique relationship between the expression of RNA splicing factors and response to the BCL2 inhibitor VEN, as well as to an inhibitor of splicing-dependent kinases, which overcomes VEN resistance [18]. Other studies showed the selective efficacy of VEN in unfit AML-MR patients under low-intensity treatment regimens [19].

**Figure 1:**
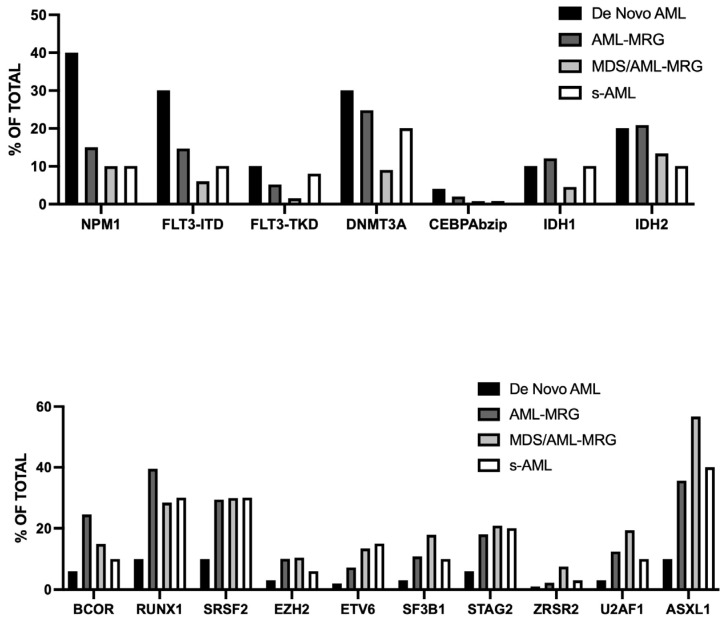
Frequency of the main gene mutations observed in AML patients subdivided into four groups: de novo AML, AML-MRG, MDS/AML-MRG, and s-AML. (**Top panel**): gene mutations (*NPM1*, *FLT3-ITD*, *FLT3-TKD*, *CEBPA^bzip^*, *IDH1*, and *IDH2*) preferentially observed in de novo AML. (**Bottom panel**): gene mutations *ASXL1*, *BCOR*, *ETV6*, *EZH2*, *RUNX1*, *SF3B1*, *STAG2*, *UAF1*, and *ZRSR2*, which are significantly associated with s-AML, MDS/AML-MRG, and AML-MRG. Original data are reported in Boertjes et al. [20].

Bortjes et al. have reported an analysis of molecular abnormalities and outcomes of a retrospective cohort of 2684 intensively treated AML patients [20]. In these patients, three subsets of AMLs harboring MRG mutations were analyzed: sAML defined as AMLs preceded by a clinically documented MDS; st-AML molecularly defined as secondary-type AML (ST-AML) with at least one mutation in *ASXL1*, *BCOR*, *ETV6*, *EZH2*, *SF3B1*, *STAG2*, *UAF1*, and *ZRSR2* (≥20% of leukemic blasts); and as-MDS-AML, molecularly defined as secondary-type MDS/AML with at least one mutation in *ASXL1*, *BCOR*, *ETV6*, *EZH2*, *SF3B1*, *STAG2*, *UAF1*, and *ZRSR2* (10–19% of leukemic blasts) [20]. The comparison of these groups of AMLs showed that mutations of *NPM1*, *FLT3-ITD*, *FLT3-TKD*, and *DNMT3A* are associated with de novo AML ontogeny, while mutations in *ASXL1*, *BCOR*, *ETV6*, *EZH2*, *RUNX1*, *SF3B1*, *STAG2*, *UAF1*, and *ZRSR2* are significantly associated with s-AML, MDS/AML-MRG, and AML-MRG [20] (Figure 1). Among these three groups of AMLs with MRG mutations, sAML had the lowest OS, while st-AML and st-MDS-AML displayed a similar OS [20]. In particular, s-AML had a 1-year-shorter OS compared to MDS/AML (45.7% vs. 72.2%) and st-AML (45.7% vs. 65.4%); however, s-AML displayed a trend toward a lower OS compared to MDS/AML (30.4% vs. 44.9%) and st-AML (30.4% vs. 40.1%) [20]. At the molecular level, st-AML and s-MDS-AML are highly comparable, except for a higher frequency of *ASXL1* mutations in MDS-AML (56.7% vs. 35.6%) and a higher occurrence of *DNMT3A* mutations in st-AML (24.9% vs. 8%) [20]. st-AMLs exhibited an OS highly comparable to that observed for adverse-risk AML, as stratified according to ELN2022 [21]. It is important to note that s-AML was not validated in current classifications. In fact, both ELN2022 and ICC2022 recognize AML following MDS as a diagnostic qualifier rather than as a separate disease entity [20].

A multicenter retrospective study of a cohort of AML, including 75 MDS-AML patients, confirmed the molecular and clinical convergence between AML-MR and MDS-AML [22]. AML-MR and MDS-AML patients exhibited overlapping molecular features, including frequent *ASXL1* and *TP53* mutations, as well as a high frequency of complex karyotypes; non-MR cases showed significantly lower frequencies of these alterations but were enriched for *NPM1* and *FLT3* mutations (25.0% and 22.7%, respectively). Both AML-MR and MDS-AML had significantly shorter OS than AMLs without MR; OS was similar for AML-MR and MDS-AML (10.3 months vs. 13.3 months, respectively) [22]. These observations suggest that MDS-AML and AML-MR form a biological continuum with shared clinical and molecular features.

Attardi and coworkers have reported an extensive characterization of 924 patients with MDS/AML (10–19% of blasts) or AML (>20% of blasts), classified according to ICC criteria [23]. This cohort included 109 patients with mutated *TP53*, 497 with MRG mutations, 93 with MR-cytogenetic abnormalities, 77 with t-AML, and 136 control AML [20]. In the group of *TP53*-mutated AML, 74 were classified as AML and 35 as MDS/AML; chromosome 17/17p abnormalities were more frequent in AML than in MDS/AML (27% vs. 8.6%), as well as *DNMT3A* mutations (24% vs. 0%) [23]. In the group with MR-cytogenetic abnormalities, 65 patients were classified as AML and 29 as MDS/AML; chromosome 5 abnormalities were less frequent in AML than in MDS/AML (23% vs. 50%), as well as del(20q) (1.5% vs. 18%) [23]. In the large group of AML patients with MRG mutations, 251 were classified as AML and 246 as MDS/AML; *FLT3*, *DNMT3A*, *CSFR3R*, *IDH1*, *IDH2*, and *BCOR* mutations were more frequent in AML than in MDS/AML, as well as the median clonal size of *STAG2* and *RUNX1* mutant alleles [23]. The groups with *TP53* mutations and with MR-related cytogenetic abnormalities have a dismal prognosis, which was comparable for both AML and MDS groups; in the group with MRG mutations, MDS/AML patients had significantly better prognoses than AML patients [23]. The study of the group of secondary AMLs (s-AML) based on clinical history (post-MDS or post-MDS/MPN), with respect to genetically defined s-AML, showed that, in all three groups of patients, no differences in outcome occurred; the only significant differences were observed for frequencies of *ASXL1* and *SF3B1* mutations, which were higher in post-MDS AML than in de novo AML (48% vs. 25% for *ASXL1* and 19.0% vs. 7.1% for *SF3B1)* [23]. The comparison of post-MDS AML with a group of patients with t-AML showed a greater frequency of *ASXL1*, *RUNX1*, *SF3B1*, *SRSF2*, *JAK2*, and *TET2* gene mutations in the former group compared to the latter [23]. A high proportion of post-MDS AMLs are classified as AML-MR, following WHO 2022 criteria, and were classified as adverse risk according to ELN 2022 criteria [23].

The results of these studies showed consistent similarity between MDS-AML and AML-MRG, suggesting a continuum from MDS/AML to AML-MR. MDS and its precursor lesions progress in a highly variable and unpredictable pattern, ranging from a slow, stable condition over many years to a rapid decline. In about 30% of cases, MDS advances into secondary AML (s-AML), which bears some of the typical molecular features of MDS. A recent longitudinal study evaluated the 6-year rate of progression of MDS and its precursor lesions, showing rates of progression of 17%, 22%, 52%, and 73% for idiopathic cytopenia of unknown significance (ICUS/IDUS), clonal cytopenia of unknown significance (CCUS), low-risk-MDS (LR-MDS), and high-risk-MDS (HR-MDS), respectively [24]. MDS and s-AML share significant genetic homology. This disease progression is a multistep process, where early driver mutations establish the initial MDS clone, and subsequent genetic events drive progression to overt leukemia. The founding early mutations typically involve RNA splicing (*SF3B1* and *SRSF2*), epigenetic regulation (*ASXL1*, *DNMT3A*, and *TET2*), or transcription factors such as *RUNX1* [25,26]. Evolution to AML is frequently driven by the acquisition of mutations in signal transduction genes, such as *FLT3*, *NRAS*, and *KIT*, or transcription factors (*RUNX1*) [26,27,28,29]. Furthermore, about 50% of cases of MDS transitioning to s-AML develop complex chromosome abnormalities, frequently involving losses or structural changes in chromosomes 5, 7, and 17 [26,27,28,29].

Genomic analyses at the single-cell level showed that mutations in transcription factor genes (such as *RUNX1* or *ETV6*) and in signaling genes (such as *FLT3*, *KRAS/NRAS*, and *PTPN11*) are typically subclonal in MDS, occurring over a “background” founding clone characterized by an epigenetic modified gene (*ASXL1*, *TET2*, and *EZH2*) and frequently accompanied by spliceosome gene mutations *(SRSF2* and *SF3B1*). Signaling gene mutations are infrequent in MDS at diagnosis, as detected by standard NGS studies; however, Guess et al., using sensitive PCR studies, showed that these mutations, at a VAF < 2%, are observed in a subset of MDS patients [26,27,28,29]. In many MDS patients, new signaling gene mutations arose concomitantly with progression to s-AML, while in other cases, pre-existing subclonal signaling mutations expanded or contracted as the blast count increased [26,27,28,29]. The presence of these subclonal signaling pathway mutations was associated with an increased risk of MDS progression, particularly in low-risk MDS patients [26,27,28,29]. Changes in the clonal architecture of progressing MDS were classified as static or dynamic, with dynamic clonal architectures having a more proliferative phenotype by blast count fold change [26,27,28,29]. Complex patterns of clonal evolution are also observed in some instances, with some clones persisting with the acquisition or loss of signaling mutations in other clones [26,27,28,29].

**Figure 2:**
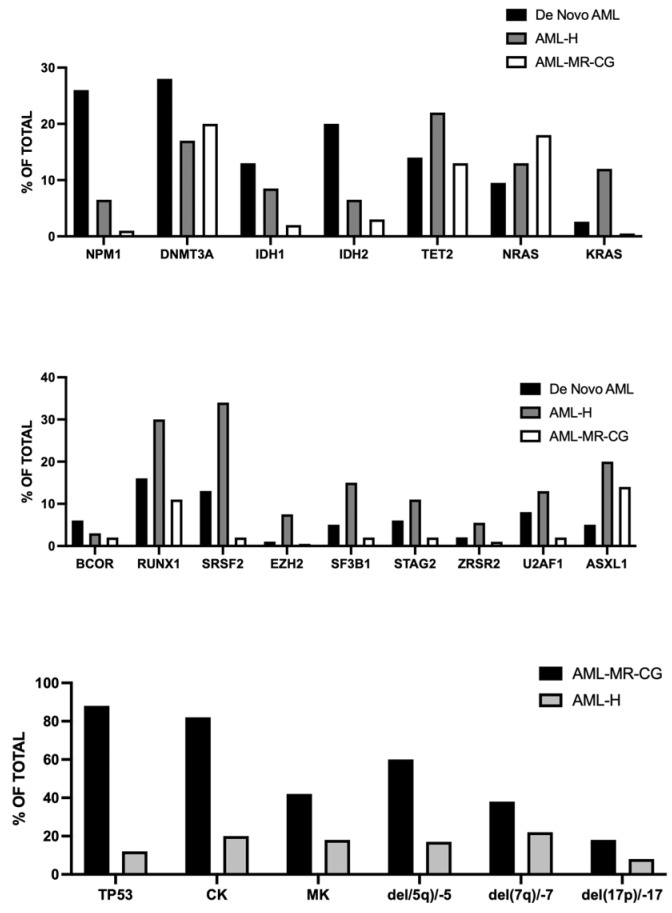
Main gene mutations in de novo AML, s-AML and AML-MR-CG (**top** and **middle** panels); main cytogenetic alterations in s-AML and AML-MR-CG (**bottom panel**). The patients were classified according to the WHO 2016 criteria as de novo AML (157 patients, without MRC), AML-H (107 patients, with a preceding documented history of MDS), and AML-MR-CG (29 patients, with MDS-related cytogenetic abnormalities). Original data are reported in McCarter et al. [30].

McCarter et al. have explored the existing interaction between AMLMRC defined on the basis of ontogeny (s-AML) and on the basis of MRG mutations [30]. Thus, they explored a group of 344 ND AML patients, who were studied at the molecular and cytogenetic levels, with detailed clinical histories. The patients were treated with intensive chemotherapy induction regimens [30]. First, these investigators carefully re-evaluated the ontogeny assignments of these patients and, according to the WHO 2016 diagnostic criteria, they defined two subgroups of AML-MRC patients: one group with a preceding documented history of MDS (MR-H), corresponding to 107 patients; a second group with MDS-related cytogenetic abnormalities (MR-CG), corresponding to 29 patients; a group of 157 patients (defined as de novo AML) with no MRC according to WHO 2016, and a group of 92 patients with t-AML [30]. The analysis of the mutational profile showed that MRG mutations were observed in 50% of MR-H, 4.5% of MR-CG, 17% of t-AML, and 28% of de novo AML patients [30] (Figure 2). Among the MR gene mutations, only *EZH2* and *SF3B1* mutations were associated with an inferior outcome in the univariate analysis. A high rate of *TP53*-mutant AMLs was observed in AML-MR-CG, and this finding was not surprising since these AMLs were selected according to the WHO criteria, which include *TP53*-mutant AMLs among AML-MR (Figure 2). It is of interest to note that AML-MR-MG cases selected according to the WHO criteria display a high rate of MDS-related chromosomal abnormalities, but only a low rate of MDS-related mutations (Figure 2). The outcomes of these AML subgroups showed that MR-H, as well as MR-CG, has a poor prognosis compared to de novo AML; de novo AML with MRG mutations has outcomes comparable to cases without MRG mutations and displays a survival profile that is intermediate between favorable and intermediate risk (ELN 2022) [30].

Other recent studies have shown the negative outcome of s-AML patients who developed AML transformed from MDS or MDS-MPN; s-AML was associated with poor outcomes irrespective of AML genomics and treatment, suggesting the necessity of its inclusion as an independent AML adverse-risk subgroup for accurate prognostication and clinical trial reporting [31].

Liu et al. have retrospectively analyzed 619 newly diagnosed AML patients, with complete cytogenetics and mutational profiling using targeted NGS [19]. For the analysis of the AML-MR subgroup, they subdivided AMLs into four subgroups: RUNX1, with *RUNX1* mutations without any other MRG mutation; MR, with any MRG mutation exclusive of *RUNX1*; RUNX1-MR, with mutations in *RUNX1* and at least one additional MRG gene; and MR-GC, with cytogenetic myelodysplasia-related mutations [32]. In this analysis, *TP53*-mutant AMLs were excluded from AML-MR and considered as an independent group. The OS of these four subgroups of AML-MR was similar; AML-MRC displayed a superior prognosis compared to the *TP53*-mutant group, but inferior to other AML diagnostic categories without MRG mutations [32]. The results of this study, as well as of other studies that evaluate *TP53*-mutant AML as a separate entity, strongly support a unified classification of AML-MR, including the different subsets of AML-MR as based on mutational profile (AML-MRG), patient history (AML-H), or cytogenetic profile (AML-CG), and support the view that AML-MR has to be considered as a unified and distinct disease entity.

## 4. MRG Mutations in FLT3-Mutant AMLs

**Figure 3:**
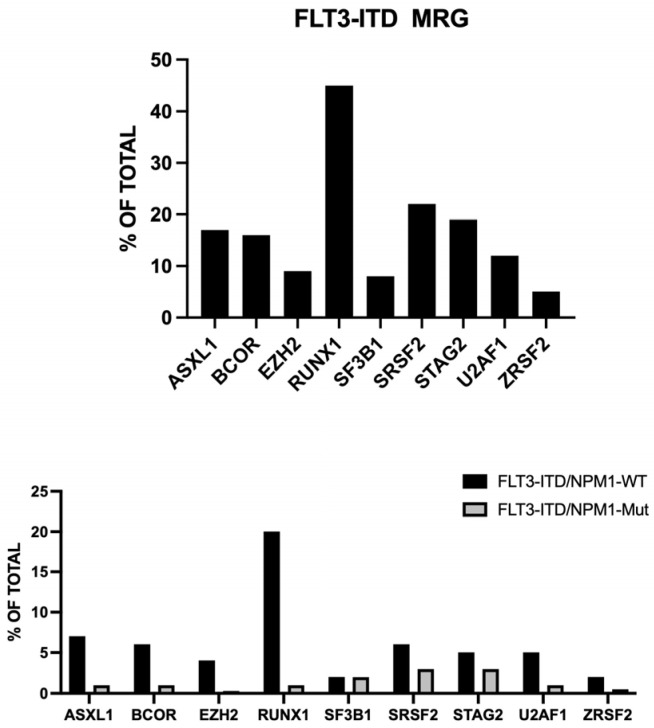
Most recurrent MRG mutations observed in *FLT3-ITD* AML patients. (**Top panel**): The frequency of MRG mutations was reported as the percentage of total MRG mutations in a cohort of 171 *FLT3-ITD* MRG patients. (**Bottom panel**): Frequency of the various MRG mutations observed in *FLT3-ITD/NPM1-WT* (371 patients) and *FLT3-ITD/MPM1^mut^* (491 patients) AMLs. Original data are reported by Mecklenbrauck et al. [33].

A recent study evaluated the prognostic impact of MRG mutations in a large cohort of 842 AML patients with *FLT3-ITD* mutations. In total, 171 (20%) of these *FLT3-ITD*-mutant AMLs display MRG mutations: 63% had only one mutation, 26% had two mutations, and 11% had three or more mutations; 57% of these patients were *NPM1*-mutated [33]. In MRG-mutated AMLs, the most common MRG mutations were *RUNX1* (45%), *SRSF2* (22%), *STAG2* (19%), and *ASXL1* (17%) [33] (Figure 3). MRG mutations occurred in 34% of *FLT3-ITD/NPM1*-WT patients and in 9% of *FLT3-ITD/NPM1^mut^* AML patients; *DNMT3A* mutations were more common in *FLT3-ITD/NPM1^mut^* AML, while *NRAS*, *ASXL1*, *BCOR*, *EZH2*, *RUNX1*, and *U2AF1* mutations were more common in the *FLT3-ITD/NPM1*-WT group [24] (Figure 3). In multivariate analysis, MRG co-mutations in the *FLT3-ITD*-mutant group did not alter outcomes and did not confer adverse prognoses in the overall *FLT3-ITD^pos^* cohort [20]. Both RFS and OS were significantly shorter in *FLT3-ITD/NPM1*-WT patients with MRG mutations compared to patients without MRG mutations; in contrast, in *FLT3-ITD/NPM1^mut^* AMLs, the presence of MRG co-mutations did not affect outcomes [33]. The *FLT3-ITD* allelic ratio did not impact the prognostic impact of MRG mutations [33]. In *FLT3-ITD/NPM1*-WT patients, the number of MRG mutations affected prognosis, since patients with a single MRG mutation had RFS and OS comparable to MRG-negative patients, while patients with two or more mutations had significantly worse RFS and OS [33].

It is of interest to note that *FLT3* mutations are rare in MDS and are acquired at the time of progression to AML [19,34,35].

## 5. MRG Mutations in NPM1-Mutant AMLs

Several studies have explored the prognostic impact of MRG mutations in *NPM1^mut^* AMLs. In this context, Eckardt et al. performed a retrospective study on 936 *NPM1^mut^* AML patients who received intensive chemotherapy treatment [36]. *NPM1^mut^* AML patients with MRG mutations are significantly older and have reduced WBC counts at initial diagnosis compared to those without MRG mutations. In total, 13.4% of these patients displayed at least one MRG mutation: *SRSF2* (5.1%), *STAG2* (3.2%), *EZH2* (2.4%), *BCOR* (1.7%), *SF3B1* (1.4%), *ASXL1* (1.3%), *ZRSR2* (0.5%), and *U2AF1* (0.4%) [36]. The rate of *FLT3-ITD* mutations was similar in both AML-MR and AMLs without MR [36]. The most recurrent mutational correlations were for co-occurring alterations of *U2AF1* and *RUNX1*, as well as *SRSF2* and *IDH2* [36]. CR rates were similar in patients with or without MRG mutations (74.4% vs. 77.7%); mRFS was 32.9 months and 24.3 months in patients without and with MRG mutations; mOS was 29.1 months and 27.2 months in patients without MRG or with MRG mutations [36]. The rate of allo-HSCT between patients without MRG mutations or with MRG mutations differed significantly regarding HSCT in the first CR (15.4% vs. 7.2%, respectively) [36]. The rate of HSCT as salvage therapy was similar in the two groups of patients [36].

Othman and coworkers confirmed these findings, reporting a 3-year OS of 67% in *NPM1^mut^* AMLs with MRG mutations compared to 65% in *NPM1^mut^* AMLs without MRG mutations [37].

However, other studies showed that MRG mutations confer a negative prognosis to *NPM1^mut^* AMLs. Thus, Chan et al. explored a group of 233 *NPM1^mut^* AMLs, 18.5% of which had MRG mutations. Patients with MRG mutations had worse OS than those without MRG mutations (15.3 months vs. 43.7 months) [38]. However, in the group of patients with MRG mutations (86% of patients were 60 years or older, compared to 52% in the group without MRG mutations), aged patients with AML-MR had significantly worse OS than aged patients without MRG mutations (12.6 months vs. 26.1 months); however, in patients younger than 60 years, there was no difference in OS between patients with or without MRG mutations [38]. MRD-negativity in *NPM1^mut^* patients predicts longer OS; the mOS of *NPM1^mut^* patients with MRG mutations achieving MRD negativity was lower than that observed for patients with *NPM1^mut^* without MRG mutations achieving MRD negativity [29]. This study suggests that the co-occurrence of MRG mutations in *NPM1^mut^* AMLs confers a significant detrimental effect on survival, which is partly related to the older age of these patients.

A more recent study further highlighted that the adverse effect of MRG mutations in *NPM1^mut^* AML patients is mainly related to their older age [39]. In fact, when stratified by age, older *NPM1^mut^* AML patients had worse OS than younger *NPM1^mut^* patients overall, although the presence of MRG mutations trended toward even worse OS in older patients [39]. In younger patients, MRG mutations are not associated with a difference in OS [39].

Cocciardi et al. reported the analysis of 568 *NPM1^mut^* AML patients; 18.1% of these patients had MRG mutations [40]. The mutated genes were *SRSF2* (7.7%), *STAG2* (4.9%), *ASXL1* (2.5%), *EZH2* (1.2%), *U2AF1* (1.2%), *ZRSR2* (1.1%), *SF3B1* (1.1%), *RUNX1* (0.7%), and *BCOR* (0.4%) [31]. *SRSF2* mutations were preferentially associated with *IDH2* and *TET2* mutations, while *STAG2* mutations were preferentially associated with signaling gene mutations (*FLT3*, *PTPN11*, *NRAS*, and *KRAS*). The frequency of MRG mutations was markedly influenced by age, being found in 8% of patients who were 18–60 years old and in 27% of patients > 60 years old [40]. VAF analysis of MRG mutations showed that these mutations represented the dominant clone over *NPM1* mutations in 76% of cases, and the co-dominant in 16% of cases. Only in 8% of cases was *NPM1* the dominant clone [40]. Restricting the analysis to 470 of these patients with a 2022 ELN favorable-risk profile, multivariable analysis for EFS identified age, *DNMT3A^R882^*, and MRG mutations as unfavorable factors; restricting the analysis to a subset of CR-CRc patients with available data on *NPM1^mut^* MRD status, MRG mutations lost their significant effect, whereas *DNMT3A^R882^* mutations maintained their unfavorable effect [40].

A recent meta-analysis evaluated the outcomes of 4363 *NPM1^mut^* AML patients, of whom 655 (15%) had co-occurring MRG mutations; the presence of MRG mutations in these patients was associated with significantly reduced mOS (hazard ratio (HR) 1.10, *p* < 0.001), shorter EFS (HR 1.43, *p* < 0.006), and a lower probability of achieving a complete remission (HR 0.94, *p* < 0.01) [41]. Subgroup analysis, limited to *NPM1^mut^* AMLs classified as favorable risk following ELN2022, confirmed the unfavorable effect of MRG mutations on OS (HR 1.34) [41]. Subgroup analysis of the most frequent co-occurring mutations, including *ASXL1*, *SRSF2*, and *STAG2*, showed that only *ASXL1* mutations were associated with inferior OS, while *SRFS2* and *STAG2* were not [41]. These findings may have important implications for the risk stratification of *NPM1^mut^* patients; in fact, in the ELN 2022 risk stratification, MRG mutations do not modify the favorable risk profile conferred by *NPM1^mut^* in the absence of *FLT3-ITD* and adverse-risk cytogenetics [41]. Patients with *NPM1^mut^* and MRG mutations but not harboring these additional high-risk features are classified as favorable risk and are not considered candidates for HSCT in CR1. However, the results of this meta-analysis suggest reconsidering this problem. In the absence of specific clinical data on these patients, the problem of selection for allo-HSCT of individual *NPM1^mut^* AML patients with MRG mutations and with favorable-risk profiles must be individualized and based on the evaluation of MRD status and on the balance of risk–benefit of an allo-HSCT [42].

Zhou et al. reported the retrospective analysis of 221 adult AML patients with a favorable-risk profile according to the ELN 2022 classification [43]. In total, 21.3% of these patients harbored MRG mutations; this subgroup of AMLs displayed a higher frequency of *TET2* and *ETV6* mutations. The presence of MRG mutations did not impact 2-year OS in the whole group of these AMLs; however, the sub-analysis of these patients according to the number of MRG mutations per patient showed that patients with two or more MRG mutations had a significantly reduced OS compared to those without MRG mutations [43]. Thus, quantification of the MRG mutation burden is required for the risk stratification of MRG-mutant AMLs with a favorable-risk profile [43].

## 6. Treatment of AML-MR

Therapy of AML-MR patients is challenging due to the limited responsiveness to current treatments and the age of the treated patients. Treatment for fit patients prioritizes intensive induction with the traditional 7 + 3 chemotherapy regimen or CPX-351, while for unfit or older patients, the standard treatment consists of lower-intensity therapy using hypomethylating agents (HMAs, azacitidine or decitabine) plus venetoclax. Allogeneic stem cell transplantation is the only curative option for these patients.

CPX-351 is a dual-drug liposomal formulation of cytarabine and daunorubicin that delivers a synergistic 5:1 drug ratio into leukemia cells to a greater extent than normal bone marrow cells. CPX-351 was approved for initial induction therapy in patients with s-AML, defined by cytogenetics, morphology, or clinical history, and t-AML based on a phase III trial that showed improved OS compared to daunorubicin and cytarabine (7 + 3 regimen) [44]. A post hoc analysis of the patients enrolled in this phase III clinical study showed that CPX-351 improved survival without pronounced myelotoxicity in patients with AML-MR mutations with respect to 7 + 3 chemotherapy (mOS of 9.7 vs. 6.8 months) [45]. CPX-351 also improved the survival of patients undergoing transplantation (2-year survival of 76% vs. 27%) [45].

Palmieri and coworkers reported the analysis of the outcomes of 403 ND AML patients with AML-MR (80.6%) or t-AML (19.4%) who received treatment with CPX-351. These patients were classified as fit or unfit for intensive chemotherapy; in the group of AML-MR patients, the CR + CRc response rate was 56.8% for fit patients and 59.2% for unfit patients [46]. However, median OS was significantly lower for unfit patients compared to fit patients (8 months vs. 18 months) [46].

The efficacy of CPX-351 was also evaluated in younger AML-MR patients. Thus, the randomized UK-NCRI AML 19 trial comparatively evaluated CPX-351 and FLAG-IDA chemotherapy regimens in 189 AML patients with adverse karyotypes and in high-risk MDS [47]. MDS-related cytogenetics was observed in 73% of these patients and AML-MR in 44% of patients [47]. The overall response rate after two courses of chemotherapy was 73% for CPX-351 and 76% for FLAG-IDA [47]. Patients with MRG mutations, but not those with MDS-related cytogenetics, had significantly longer mOS with CPX-351 than with FLAG-IDA (38.4 months vs. 16.3 months, respectively) [47].

Other studies have explored the safety and efficacy of venetoclax + HMAs in AML-MR patients. Shimony and coworkers analyzed a group of 314 ND AML patients treated with HMA alone or VEN + HMA; the response to treatment was evaluated in three groups of patients subdivided as AML with MR, de novo AML without MR, and *TP53*-mutant AML. In the whole AML cohort, Ven + HMA treatment was found to be better than HMA treatment alone (mOS of 9.9 months vs. 7.4 months, respectively). The analysis of the outcomes in the three groups of patients showed that the benefit from the Ven + HMA treatment was restricted to the AML-MR group; in fact, in AML-MR patients, mOS was 14.1 months with Ven + HMA compared to 6.9 months with HMA; in the de novo AML group, mOS was 13.2 months with Ven + HMA and 10.3 months with HMA; and in the *TP53* group, mOS was 5.7 months with Ven + HMA and 6.1 months with HMA [48]. Only in the group of AML-MR patients did Ven + HMA significantly increase the rate of patients who underwent allo-HSCT (24% with Ven + HMA compared to 6% with HMA) [48].

In a second study, Shimony et al. compared the response of 395 AML-MR patients to three different induction regimens: daunorubicin and cytarabine (7 + 3 regimen), liposomal daunorubicin and cytarabine (CPX-351), and Ven + HMA [35]. Mean OS was comparable in these three groups of patients [49]. In multivariable analysis, Ven + HMA was associated with better OS than 7 + 3 chemotherapy, whereas CPX-351 was not better than 7 + 3 chemotherapy [49]. NRAS/KRAS co-mutations had a detrimental effect on the response to Ven + HMA and CPX-351 [49]. Pairwise comparisons showed that VEN + HMA was associated with improved OS vs. 7 + 3 chemotherapy in patients with *SF3B1* mutations and improved OS vs. CPX-351 in patients with RNA splicing factor mutations [49]. In line with this study, Fathima and coworkers retrospectively analyzed 600 ND AML patients treated with Ven + HMA or CPX-351 and observed that, in AML-MR patients, the response rate and mOS were similar for patients treated with CPX-351 or Ven + HMA (CR + CRc of 60% vs. 63%; mOS of 10 months vs. 14 months, respectively); OS was superior with Ven + HMA in post-MDS AML patients (15 months vs. 8 months, respectively) and with CPX-351 in patients with *SF3B1* mutations (not reached vs. 14 months, respectively) [50].

A recent real-world study evaluated 235 adult AML patients diagnosed with AML-MR and stratified by AML-MR subgroup based on cytogenetics (AML-MRc), molecular (AML-MRm), and antecedent hematological malignancy (AML-AHD). The overall CR + CRc rate was 52%, with no difference by age. The highest response rates were observed among AML-MRm (CR + CRc 57%). In total, 55% of participants underwent allo-HSCT. Younger AML-MTm patients had longer mOS compared to older patients (38.0 vs. 19.5 months, respectively) [51].

Ikoma and coworkers explored a group of 228 AML patients, including 51.8% of cases of AML-MR, treated with Ven + HMA [52]. The analysis of survival curves showed that AML-MR patients displayed a survival profile comparable to that observed for patients defined with a favorable-risk profile [52]. According to these findings, it was concluded that MRG mutations predict a favorable response to Ven + HMA [52].

The encouraging data derived from ongoing clinical studies of intensive chemotherapy with Ven for patients with ND AML have supported the evaluation of the combination of CPX-351 with Ven in ND AML patients eligible for intensive chemotherapy. Thus, a phase II clinical study reported the evaluation of CPX-351 in 17 ND AML patients with a median age of 59 years, 71% with s-AML and 59% with MRG mutations [19]. The overall response rate was 82%, with a CRc rate of 72%; AML-MR patients had an ORR of 100%, with a CRc rate of 90% [53]. Patients with MRG mutations had a median OS of 17.9 months compared to 5.9 months in those without MRG mutations [53]. A total of 86% of the responding patients proceeded to allo-HSCT [53].

A recent study reported the results of a phase II study of SLS009 in combination with azacitidine and venetoclax for relapsed/refractory AML-MR after prior venetoclax treatment [54]. SLS009 is a highly selective cyclin-dependent kinase-9 (CDK9) inhibitor that leads to transcriptional suppression of the antiapoptotic protein MCL-1. Three cohorts of patients were enrolled: R/R AML patients after prior venetoclax treatment; R/R AML-MR patients with *ASXL1* mutations after prior venetoclax treatment; and AML-MR patients without *ASXL1* mutations after prior venetoclax-based treatment [54]. Among the 36 patients with AML-MR, 44% of patients achieved an overall response, with 29% CR; response rates appeared to be correlated with the median number of prior lines of therapy [54].

A large majority of AML-MR patients are considered high-risk AML patients according to the 2022 European Leukemia Net (ELN) risk stratification; for these patients, allogeneic hematopoietic stem cell transplantation (allo-HSCT) is the recommended post-remission strategy in transplant-eligible patients in their first complete remission (CR1) [55]. AML-MR patients without *TP53* mutations show allo-HSCT outcomes comparable to those observed for intermediate-risk AMLs as defined by ELN 2022, while patients harboring *TP53* mutations show a distinctive, poor outcome. In this context, Esteve et al. reported the results of a retrospective analysis on 1152 AML patients who underwent allo-HSCT in their first CR1: 33% with AML-MRG, 21% with AML-MR-CG, 17% with *TP53*-mut non-CK, and 28% with *TP53*-mut CK [55]. Outcomes varied consistently in these four groups of patients with an increasing relapse incidence (20%, 29%, 44%, and 57% at 2 years), decreasing LFS (60%, 44%, 40%, and 20% at 2 years), overall survival (65%, 60%, 47%, and 24% at 2 years), and RFS (47%, 39%, 32%, and 13% at 2 years) in AML-MRG, AML-MR-CG, *TP53*-mut non-CK, and *TP53*-mut CK patients, respectively [55].

Ngo et al. reported the outcomes of 141 AML-MR patients treated with either intensive chemotherapy (47 patients) or venetoclax-based regimens (94 patients); 23 patients (49%) treated with intensive chemotherapy and 14 patients (15%) treated with venetoclax underwent allo-HSCT. Patients who underwent HSCT had longer survival and longer-term disease control compared to those not transplanted; the outcomes for transplanted patients were similar for patients receiving intensive or non-intensive induction treatment [56].

## 7. Conclusions

WHO^5th^ and ICC^2022^ both include AML-MR as a new diagnostic entity that incorporates the mutation profile. Although the diagnostic criteria proposed by WHO^5th^ and ICC^2022^ overlap, some notable differences are present, including the inclusion of *RUNX1* in the AML-MR gene list (only for ICC^2022^), the evaluation of AML-MR while considering gene mutations or cytogenetic profiles as distinct entities, and the inclusion or exclusion of AML with *TP53* mutations. An additional element of discrepancy is also related to the impact of AML ontogeny, and consequently the consideration, as a unique entity, of AML patients with a history of antecedent MDS or MDS/MPN.

The studies carried out in the last few years strongly suggest that *RUNX1* should be included in the MR gene list; that *TP53*-mutant AML should be excluded from AML-MR and considered as a separate entity; and that AML-MR should include, as a unique entity, AMLs with MRG mutations, AMLs with MR-CGAs, and AMLs with an antecedent history of MDS or MDS/MPN. This unified classification of AML-MR will facilitate the planning of dedicated clinical trials, their harmonization, and their comparative evaluation.

## Figures and Tables

**Table 1:** Classification of AML-MR following WHO 5th ed., 2022, and ICC, 2022.

Feature/Criteria	WHO Classification (5th Edition, 2022)	International Consensus Classification (ICC, 2022)
Category Name	AML myelodysplasia-related (AML-MR)	Divided into: AML with MDS-related gene mutations, AML with MDS-related cytogenetic abnormalities
Myeloblast Threshold	≥20% blast threshold requirement for the vast majority of AML-MR cases to qualify as AML	≥20% blast threshold, but cases with 10–19% blasts are placed into a distinct MDT/AML category
Antecedent Clinical History	A prior history of MDS or MDS/MPN is incorporated directly into AML-MR-defining criteria	Clinical history, such as antecedent MDS, is considered as a “diagnostic qualifier” rather than a strict criterion for AML-MR
Genetic Mutations	Defined by specific MDS-related mutations (*ASXL1*, *BCOR*, *EZH2*, *SF3B1*, *SRSF2*, *STAG2*, *U2AF1*, *ZSZR2*)	Similar gene mutations, but also includes *RUNX1* mutations
TP53 Mutations	*TP53* mutations are included within the broader AML-MR mutation profile	AML with mutated *TP53* is established as a separate, very high risk, independent entity that takes precedence over other AML subtypes
Cytogenetic Criteria	Defined by specific MDS-related cytogenetic abnormalities (e.g., 5//del(5q), −7/del(7q), −13/del(13q), i(17q))	Similar cytogenetic abnormalities, but does not allow classification if a *TP53* mutation is present

## Data Availability

No new data were created or analyzed in this study. Data sharing is not applicable to this article.
